# Water cycle changes in reanalyses: a complementary framework

**DOI:** 10.1038/s41598-023-31873-5

**Published:** 2023-03-23

**Authors:** Mijael Rodrigo Vargas Godoy, Yannis Markonis

**Affiliations:** Faculty of Environmental Sciences, Czech University of Life Sciences Prague, Kamýcká 129, 165 00 Praha-Suchdol, Czech Republic

**Keywords:** Hydrology, Hydrology

## Abstract

Climate reanalyses complement traditional surface-based measurements and offer unprecedented coverage over previously inaccessible or unmonitored regions. Even though these have improved the quantification of the global water cycle, their varying performances and uncertainties limit their applicability. Herein, we discuss how a framework encompassing precipitation, evaporation, their difference, and their sum could further constrain uncertainty by unveiling discrepancies otherwise overlooked. Ahead, we physically define precipitation plus evaporation to describe the global water cycle fluxes in four reanalysis data sets (20CR v3, ERA-20C, ERA5, and NCEP1). Among them, we observe four different responses to the temperature increase between 1950–2010, with ERA5 showing the best agreement with the water cycle acceleration hypothesis. Our results show that implementing the framework proposed can improve the evaluation of reanalyses’ performance and enhance our understanding of the water cycle changes on a global scale.

## Introduction

Understanding the global water cycle and its balance is crucial for Earth system science and climate change studies. To assess the water cycle at multiple spatiotemporal scales, we observe and measure the fluxes and storage that comprise its budget. The data sources we rely on for such research have continuously evolved, even though they remain thwarted by uncertainty^1^. Ground observations are regarded as the closest measurements to the actual values, but we still lack a comprehensive global network. E.g., the Global Precipitation Climatology Centre (GPCC)^2^, currently the most extensive gauge network available, represents only about 1% of the Earth’s surface (assuming a 5 km non-overlapping radius per gauge)^3^. While a kindred initiative for evaporation exists (FLUXNET)^4^, evaporation is more commonly derived from atmospheric moisture and precipitation measurements than directly observed. Satellite remote sensing data complement ground measurements by offering observational coverage on a global scale. Its record, nonetheless, is too short to assess long-term changes of water cycle fluxes.

Reanalysis products assimilate observational data into general circulation models or, most recently, earth system models. Broadly speaking, assimilation algorithms recursively combine the model outputs and observations within a Bayesian statistical framework. As a result, the physical conservation principles are overstepped, which is reflected in substantial variability compared to that of other data sources^5^. Since their early implementation, concerns about the reliability of reanalyses to assess the global water cycle have been raised^6^. Despite individual advancements in model simulations as well as assimilation algorithms, it has been reported that moisture recycling is too large and its lifetime too short^7^. Regardless, reanalyses remain one of the most comprehensive data sources because of their high spatiotemporal resolution and capability to switch anthropogenic forcing on and off^8^.

Therefore, it is no surprise that they have been used in the estimation of water cycle fluxes and their changes. A prime example is the work of Trenberth et al.^9^, therein the authors describe the global water budget and its annual cycle. While the authors have reservations about reanalysis-based results, they acknowledge the potential for in-depth analysis using reanalyses. It is worth mentioning that the work of Trenberth et al.^9^ is in high regard by the scientific community, and their results are often used to benchmark more recent studies. During the last decade, global water cycle research has explored multi-source data integration, exploiting observational and reanalysis data availability. Specifics between methods vary, but, in general, data sets are merged in three steps: initial assessment of the data, integration of the products, and budget closure post-processing.

Some examples at the global scale are the works of Rodell et al.^10^ and Zhang et al.^11^, where the authors use multiple reanalysis evaporation/evapotranspiration products to assess the water cycle and budget closure. Rodell et al.^10^ relied on reanalyses at various other stages of their analysis, such as data sources for other variables (e.g., atmospheric convergence, wind, and surface pressure), to downscale observations, and to fill data gaps. The authors convey that independent reanalysis estimates enable assessing uncertainty with a higher degree of confidence. Zhang et al.^11^ studied the influence of data sources on water budget closure experiments and concluded that integrating reanalysis data reduces the non-closure errors significantly. Yet, further efforts are needed to understand the discrepancies among different data sources.

In this study, the representation of global water cycle changes is assessed in four reanalysis data sets for the first time. To achieve it, we physically define precipitation plus evaporation to unveil hidden details that have been overlooked due to the lack of a more exhaustive framework. We assessed the following data sets: 20CR v3^12^, ERA-20C^13^, ERA5^14^, and NCEP1^15^. First, we compare the reanalyses using ground-based data as a reference GPCP v2.3^16^ and HadCRU5T5^17^ for precipitation and temperature, respectively. Then, we inspect $P - E$ to check for budget closure, and we evaluate the $P + E$ behavior in terms of hydrological sensitivity. Next, we present the application of $P + E$ in a framework that describes the changes in the water cycle. We achieve this by exploring the changes in atmospheric water fluxes and storage redistribution between land-ocean and the atmosphere. Finally, we discuss the possible connotations of the findings regarding $P + E$ and its application as a performance metric for reanalysis data.

## The physical basis

Over land, the net water flux into the surface, a vital aspect of the water cycle for human society, is described by the difference between precipitation and evaporation ($P - E$). Thus, $P - E$ characterizes atmosphere-land surface interactions and represents the maximum available renewable freshwater^18^. Analogously, evaporation minus precipitation ($E - P$) determines the surface salinity of the ocean, which helps determine the stability of the water column^19^. There was a consensus that as precipitation increases overland, so does evaporation over the oceans to balance the global water cycle^20^. Nonetheless, it has recently become evident that there are contrasting responses between the terrestrial and oceanic water cycles^21,22^. Furthermore, at the regional scale moisture convergence can increase precipitation^23^. Assuming radiation is not limiting, evapotranspiration will be equally enhanced. On the one hand, $P - E$ would suggest no change in the hydrological cycle, while, on the other hand, the increase in $P + E$ would correctly indicate that the water cycle is indeed changing, with more water being circulated in total through the surface-atmosphere continuum.

Huntington et al.^24^ have already shown that the sum of precipitation and evapotranspiration can be adequately applied to quantify the changes in the terrestrial portion of the water cycle. We argue that this approach can be extended to the description of the whole water cycle because $P + E$ has a robust physical meaning; it describes the total flux of water exchanged between the atmosphere and the surface. Furthermore, like the human heart, the Earth cycles far more water through the atmosphere than its holding capacity. In this manner, it would make sense to also look into the addition of fluxes rather than only their difference when assessing the global water cycle intensification. The proposed framework is based on quantifying precipitation, evaporation, their difference, and their sum. The latter, precipitation plus evaporation, is mathematically complementary to the widely used $P - E$ metric. Nonetheless, math alone does not suffice to improve our understanding of the global water cycle. Thus, we will define $P + E$ from a mass balance and a kinematic perspective.

### Water cycle budget

The global water cycle’s mass balance is expressed with the water budget equation:

1 $$\begin{aligned} P + Q_{\text {in}} = E + Q_{\text {out}} + \Delta S \end{aligned}$$

where *P* is precipitation, $Q_{in}$ is water flow into the Earth, *E* is evaporation (since we are at the global scale we will refer to it simply as evaporation for brevity, but we acknowledge it encompasses evaporation from soils, surface-water bodies, and plants), $\Delta S$ is water storage change in the land-ocean continuum (biological water, fresh lakes, ice, nonrenewable groundwater, oceans, permafrost, reservoirs, renewable groundwater, rivers, saline lakes, seasonal snow, soil moisture, and wetlands), and $Q_{out}$ is water flow out of the Earth. All terms are averaged globally over a fixed time period (e.g., mm/year). At the global scale, due to Earth’s gravity and temperature, water inflow or outflow leaking between the atmosphere and outer space is negligible compared with precipitation and evaporation and water storage change. Consequently, $Q_{in}\rightarrow 0$ and $Q_{out}\rightarrow 0$ leaving us with:

2 $$\begin{aligned} \Delta S = P - E \end{aligned}$$

where $\Delta S$ represents a storage redistribution from the atmosphere towards the land-ocean continuum (positive), from the land-ocean continuum towards the atmosphere (negative), or steady state equilibrium (zero). Now, we define global water cycle intensity as:

3 $$\begin{aligned} \text {GWCI} = P + E \end{aligned}$$

In this manner, intensity is defined as the total total flux of water exchanged between the atmosphere and the land-ocean continuum. This definition is in line with previous formulations in the literature^24,25^. Furthermore, different ways to integrate precipitation and evaporation to describe the hydroclimatic regime have been in use for over half a century now (e.g., Budyko curve^26^).

### Water cycle kinematics

As established above, precipitation plus evaporation describes the water cycle intensity from a mass balance perspective by quantifying the total flux of water exchanged between the atmosphere and the land-ocean continuum. If we describe these atmospheric water fluxes from a kinematic perspective, we have two velocity vectors:

4 $$\begin{aligned} \begin{aligned} {\vec {P}}_{lon, lat}&={\textbf{P}} (x, y, z)\\ {\vec {E}}_{lon, lat}&={\textbf{E}} (x, y, z) \end{aligned} \end{aligned}$$

where, at any location on Earth’s surface, ${\vec {P}}_{lon, lat}$ is the precipitation vector with magnitude **P** and ${\vec {E}}_{lon, lat}$ is the evaporation vector with magnitude **E**. These velocities are parallel to each other but are oriented in opposite directions. We define the direction from the atmosphere to the surface as positive and the opposite (from the surface to the atmosphere) as negative, then:

5 $$\begin{aligned} \begin{aligned} {\vec {P}}_{lon, lat}&={\textbf{P}} (0\hat{i}, 0\hat{j}, 1\hat{k})\\ {\vec {E}}_{lon, lat}&={\textbf{E}} (0\hat{i}, 0\hat{j}, -1\hat{k}) \end{aligned} \end{aligned}$$

Precipitation and evaporation are heavily intertwined through moisture recycling. Therefore, we could characterize their interdependence relationship by defining the velocity of the global water cycle as the Newtonian relative velocity of precipitation with respect to evaporation:

6 $$\begin{aligned} \begin{aligned} \overrightarrow{GWC}_{lon, lat}&= {\vec {P}}_{lon, lat} - {\vec {E}}_{lon, lat}\\&={\textbf{P}} (0\hat{i}, 0\hat{j}, 1\hat{k}) - {\textbf{E}} (0\hat{i}, 0\hat{j}, -1\hat{k})\\&=(0 - 0)\hat{i} + (0 - 0)\hat{j} + ({\textbf{P}} - (-{\textbf{E}}))\hat{k}\\&=0\hat{i} + 0\hat{j} + ({\textbf{P}} + {\textbf{E}})\hat{k}\\&=({\textbf{P}} + {\textbf{E}}) (0\hat{i}, 0\hat{j}, 1\hat{k})\\ \end{aligned} \end{aligned}$$

where **(P + E)** is the magnitude of global water cycle velocity. Hence, we can safely ascertain that assessing changes in $P + E$ refers to acceleration or deceleration of the global water cycle.

## The precipitation–evaporation space

**Figure 1:**
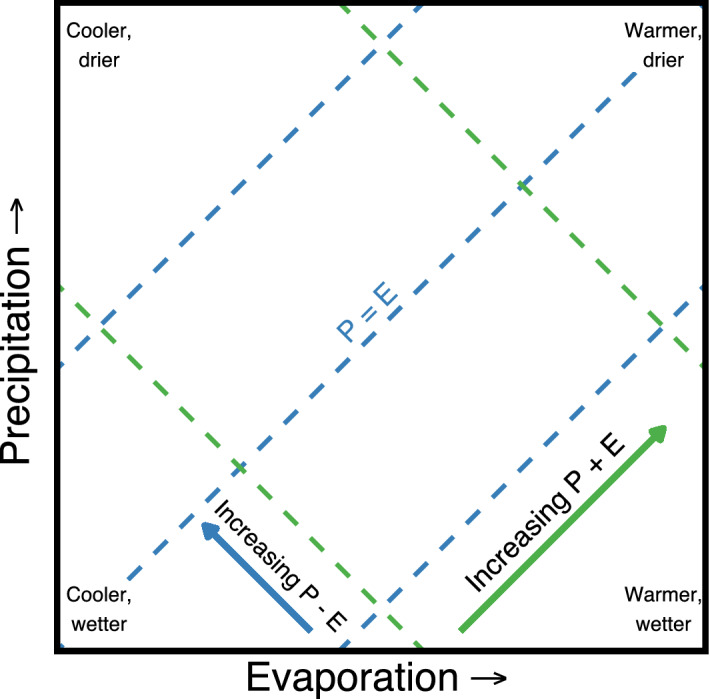
The global water cycle regime in the precipitation–evaporation space. Vectors represent water cycle changes, where *P* is precipitation, and *E* is evaporation. Contours of equal $P - E$ (no change in water cycle storage) are shown as blue dashed lines, and movement across these lines (blue vector) describe changes in water cycle storage. Contours of equal $P + E$ (no change in water cycle intensity) are shown as green dashed lines, and movement across these lines (green vector) describe changes in water cycle intensity.

Including precipitation, evaporation, their difference, and their sum provides a synthesized visual of the overall response of the water cycle to global warming. The global water cycle regimes in this framework would be described in the precipitation–evaporation space by their precipitation and evaporation coordinates, and vectors represent changes between two periods (Fig. 1). By transforming the changes in the relationship of *P* and *E* to changes in $P - E$ and $P + E$, we can describe the water cycle dynamics in terms of atmospheric water storage and fluxes correspondingly. Precipitation and evaporation may increase, decrease, or remain constant. From equation (2), changes in atmospheric water storage ($P - E$) shown as blue contours are planes that increase from the bottom right (wetter) to the top left corner (drier). It is important to note that Huntington et al.^24^ focused on terrestrial water storage, as such, the directions for drier and wetter are reversed therein. From equation (3), water cycle acceleration ($P + E$) is a plane shown as green contours that increases from the bottom left (cooler) to the top right (warmer). $P - E$ is negative to the right of the identity diagonal, zero along this line, and positive to the left of the line. At the global scale, negative values describe an increase in atmospheric water storage (wetter), positive values describe an increase in land-ocean water storage (drier), and zero describes steady-state equilibrium. $P + E$ increases describe shifts from cooler regimes into warmer ones.

## Results

### Climate reanalyses

**Figure 2:**
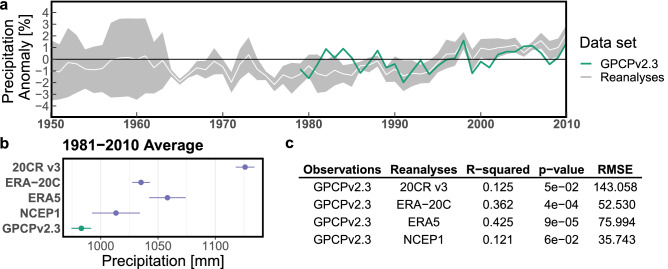
Benchmarking global spatial weighted average values of reanalysis precipitation compared to GPCP v2.3 as the observation-based reference. (**a**) Precipitation anomalies annual time series between 1950–2010 (common period between all reanalyses), spread of reanalysis estimates is shown in gray and their mean in white, GPCP v2.3 is shown in turquoise. (**b**) The 30-year average for the data sets compared, reanalysis estimates are shown in violet and GPCP v2.3 in turquoise. (**c**) Summary statistics of linear correlation between reanalysis products and GPCP v2.3 annual time series.

**Figure 3:**
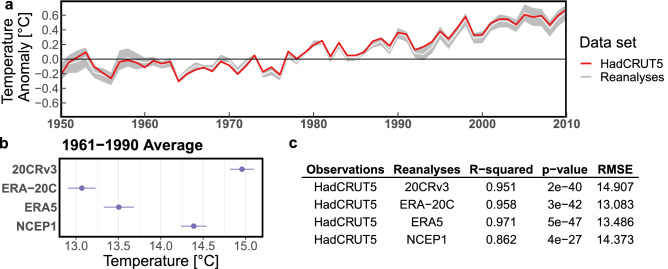
Benchmarking global spatial weighted average values of reanalysis temperature compared to HadCRUT5 as the observation-based reference. (**a**) Temperature anomalies annual time series between 1950–2010 (common period between all reanalyses), spread of reanalysis estimates is shown in gray and their mean in white, HadCRUT5 is shown in red. (**b**) The 30-year average for the data sets compared, reanalysis estimates are shown in violet (HadCRUT5 is not available). (**c**) Summary statistics of linear correlation between reanalysis products and HadCRUT5 annual time series.

Our analyses, taken together, show the potential of precipitation plus evaporation to assess reanalysis data and complement water cycle changes research. We start by exploring precipitation and temperature as portrayed in reanalyses with GPCP v2.3 and HadCRUT5 as the existing references. The variability from reanalysis precipitation becomes readily visible by the wide spread of values (Fig. 2a). We observe an abrupt reduction in reanalysis precipitation variance after the mid-1960s (narrowing of the gray area; Fig. 2a), coinciding with the satellite era’s beginning. To a greater or lesser extent, all reanalyses products overestimate precipitation, with NCEP1 having its 30-year average closest to GPCP v2.3 (Fig. 2b). Nevertheless, ECMWF reanalyses perform better than the 20CR v3 and NCEP1 (0.4 vs. 0.1 R-squared; Fig. 2c). Regarding temperature, there is considerably less variability among reanalyses and no visible abrupt changes in said behavior (Fig. 3a). Concurrently, temperature in reanalyses is centered around the $14\, ^{\circ }{\text{ C }}$ average (Fig. 3b). Furthermore, all reanalysis products exhibit a strong and statistically significant correlation to HadCRUT5 (R-squared $\gtrsim 0.9$; Fig. 3c). Overall, ERA5, with the highest R-squared values, most comprehensively captures both precipitation (R-squared 0.43) and temperature (R-squared 0.97) changes among the four reanalyses. While no direct assessment of reanalysis evaporation is possible due to the lack of observation-based reference data, and despite the general biases reported above, it is feasible to rely on reanalyses to assess global water cycle changes based on their performance versus precipitation and temperature observations.

**Figure 4:**
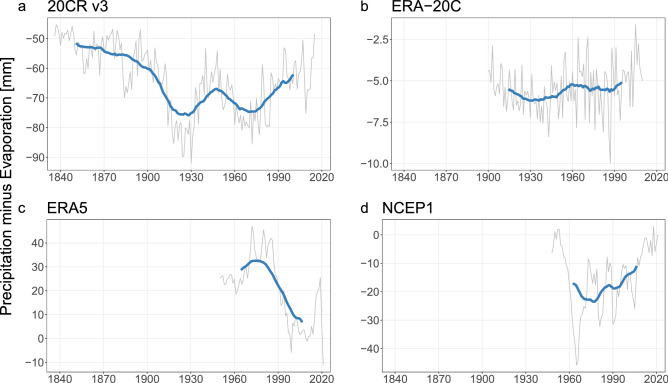
Global spatial weighted average of annual total precipitation minus evaporation in mm as depicted in four reanalysis data sets for their respective available record. Annual values are shown in gray. 30-year moving average values are shown in blue.

Atmospheric water residence time is circa nine days, and as previously stated, this lifetime is underestimated in reanalyses. Thus at annual or longer time steps, what goes into the atmosphere as evaporation has to equal what comes out as precipitation. Due to the assimilation algorithms and systematic uncertainty in reanalyses, we expected budget non-closure to some extent. However, it was surprising that even the 30-year moving average of $P - E$ in reanalyses is not steady (Fig. 4). A gripping behavior in both ongoing reanalyses, i.e., ERA5 (Fig. 4c) and NCEP1 (Fig. 4d), is that the $P - E$ trend appears to be directed towards 0 mm/year ($P = E$). In the case of the long-term reanalyses, we found opposing conducts. ERA-20C has the “flattest” $P - E$ mean at approximately $-5.5$ mm/year (Fig. 4b). On the other hand, the 20CR v3 has considerably more variability and the highest $P - E$ absolute values (Fig. 4a). A particular characteristic of 20CR v3 $P - E$ is that it seems to exhibit two regimes, one before 1900 centered around $-54$ mm/year and the second from 1900 onwards centered around $-69$ mm/year.

### Water cycle changes

**Figure 5:**
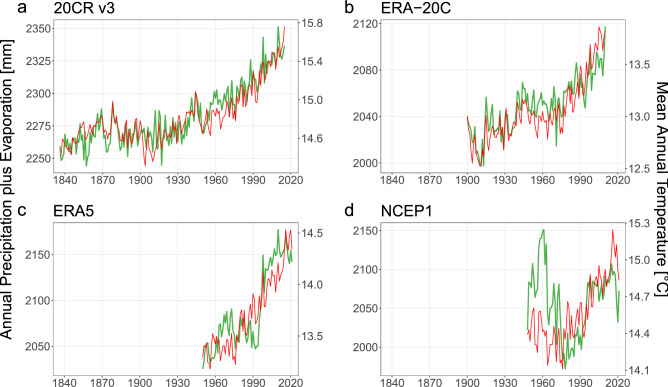
Global spatial weighted average of annual total precipitation plus evaporation and annual mean temperature in four reanalysis data sets for their respective available record. Precipitation plus evaporation in mm is shown in green. Temperature in $^{\circ }{\text{ C }}$ is shown in red.

The physical soundness of the $P + E$ metric becomes readily visible by the superimposition of the annual mean global temperature and the annual total global $P + E$ of the four reanalysis data sets (Fig. 5). Their coupling is statistically supported by quantifying the linear relationship between these variables (Table 1). The dominant behavior in the long-term relationships reports two common markers: a strong $P + E$ correlation (R-squared $\approx 0.8$; Fig. 5a–c), and an apparent decoupling between $P + E$ and temperature around the 1960s. We observe particular traits for ERA5 and NCEP1. ERA5 shows a moderate $P - E$ correlation (R-squared $= 0.39$). NCEP1, not resembling the other three data sets, has a higher correlation for the difference than the sum of precipitation and evaporation(0.18 vs 0.12 R-squared). Moreover, the coupling between $P + E$ and temperature occurs only after the mid-1970s (Fig. 5d). The robust performance of $P + E$ as a metric to substantiate the relationship between atmospheric water fluxes and temperature carries from the long-term onto the year-to-year variability (Table 1). Estimating the annual differences, we now observe a homogeneous behavior in all the reanalyses data sets with moderate $\delta (P + E)$ correlation (R-squared between 0.2–0.4) and no $\delta (P - E)$ correlation (R-squared $\le 0.02$). This independence in $\delta (P - E)$ imply that the correlation observed between $P - E$ and temperature was due to the long-term trends, while $P + E$ correlates both to short-term and long-term temperature variability.

**Table 1:** Linear relationship between global spatial weighted average of total atmospheric water fluxes and mean temperature, where *P* is precipitation, *E* is evaporation, and *T* is temperature. Long-term columns report the correlation between the annual values (i.e., $(P \pm E)$ vs. *T*). Year-to-year columns report the correlation between the annual consecutive differences (i.e., $\delta (P \pm E)$ vs. $\delta T$).

Reanalysis	Long-term				Year-to-year			
	$P + E$		$P - E$		$\delta (P + E)$		$\delta (P - E)$	
	R$^{2}$	p-value	R$^{2}$	p-value	R$^{2}$	p-value	R$^{2}$	p-value
20CR v3	0.82	$< 2\times 10^{-16}$	0.01	0.1	0.19	$1\times 10^{-9}$	$2\times 10^{-4}$	0.9
ERA-20C	0.80	$< 2\times 10^{-16}$	0.06	$1\times 10^{-2}$	0.37	$2\times 10^{-12}$	0.02	0.2
ERA5	0.75	$< 2\times 10^{-16}$	0.39	$3\times 10^{-9}$	0.35	$3\times 10^{-8}$	0.02	0.2
NCEP1	0.12	$2\times 10^{-3}$	0.18	$2\times 10^{-4}$	0.22	$2\times 10^{-5}$	$4\times 10^{-3}$	0.6

Thermodynamics, Clausius-Clapeyron scaling in particular, determine the relationship between atmospheric water vapor and temperature. However, it is the Earth’s energy balance that governs global precipitation and evaporation, and constraining the hydrological sensitivity^27^. The hydrological sensitivity, defined by the increase in global mean precipitation (or evaporation) for a given change in global mean temperature, has been estimated at $2.1{-}3.1\;\%/^{\circ }{\text{ C }}$^28^. Consequently, $P + E$ should also increase at approximately 2–$3\;\%/^{\circ }{\text{ C }}$. To validate our hypothesis, we looked into the slopes of linear regression fits between $P + E$, *P*, *E*, and temperature (Table 2). We validated the anticipated increases for $P + E$ except for ERA5, which had a rate of $5.0\pm 0.3\;\%/^{\circ }{\text{ C }}$, but also a rather high evaporation increase of $6.2\pm 0.4\;\%/^{\circ }{\text{ C }}$. R-squared offers some insight about the proportion of variance in $P + E$, *P*, and *E* that can be explained by temperature. Interestingly, evaporation has the lowest correlation to temperature across all reanalyses. 20CR v3 and ERA5 have higher R-squared values for $P + E$ than for *P*, with differences of 0.12 and 0.09, respectively. In contrast, ERA-20C and NCEP1 have higher R-squared values for precipitation (differences of 0.01 and 0.05). Note that while precipitation has a higher R-squared for ERA-20C and NCEP1, the difference is one order of magnitude smaller than those whose $P + E$ has a higher R-squared (20CR v3 and ERA5). These results demonstrate a good coupling between $P + E$ and hydrological sensitivity.

**Table 2:** Linear relationship between global spatial weighted average of total atmospheric water fluxes and mean temperature, where *P* is precipitation, *E* is evaporation, and *T* is temperature. $P + E$ columns report the correlation ($(P + E)$ vs. *T*). *P* columns report the correlation (*P* vs. *T*). *E* columns report the correlation (*E* vs. *T*). Slopes are in %$/^{\circ }$C, where the reference for atmospheric flux percentage change and temperature anomaly is their 1981–2010 average. RSE is Residual Standard Error.

Reanalysis	$P + E$			*P*			*E*		
	Slope (%$/^{\circ }$C)	RSE	R$^{2}$	Slope (%$/^{\circ }$C)	RSE	R$^{2}$	Slope (%$/^{\circ }$C)	RSE	R$^{2}$
20CR v3	$3.2 \pm 0.1$	0.42	0.82	$3.1 \pm 0.2$	0.58	0.7	$3.3 \pm 0.2$	0.69	0.65
ERA-20C	$3.3 \pm 0.2$	0.5	0.8	$3.3 \pm 0.2$	0.49	0.81	$3.2 \pm 0.2$	0.52	0.78
ERA5	$5.0 \pm 0.3$	1.05	0.75	$3.8 \pm 0.3$	1	0.66	$6.2 \pm 0.4$	1.32	0.74
NCEP1	$2.8 \pm 0.9$	2.01	0.12	$4 \pm 1$	2.18	0.17	$1.9 \pm 0.9$	1.95	0.06

**Figure 6:**
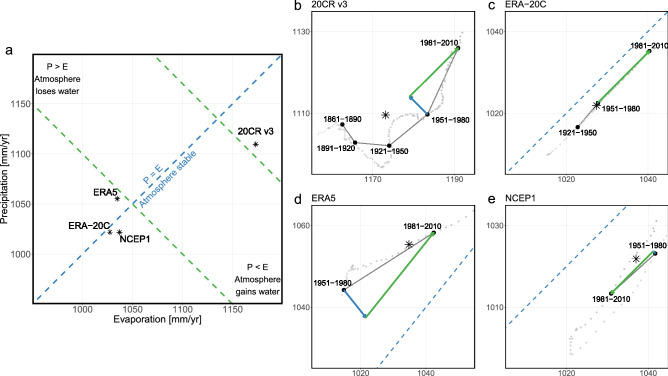
The precipitation–evaporation space graphical framework for the assessment of global water cycle changes. *P* and *E* are global total precipitation and evaporation in mm/year. Contour of $P = E$ is shown as a blue dashed line (stable atmosphere). Contours of equal $P + E$ are shown as green dashed lines (equal water cycle intensity). Changes in $P - E$ and $P + E$ are shown as blue and green vectors correspondingly. Light gray points show the 30-year moving average trajectory, black points mark the labeled 30-year period of interest, and stars mark the position of the average for the full record of each reanalyses. I.e., 1836–2015 average for 20CR v3, 1900–2010 average for ERA-20C, 1950–2020 average for ERA5, and 1948–2020 average for NCEP1. (**a**) Relative position of reanalyses with respect to each other in the precipitation–evaporation space. (**b**) Zoomed in panel on the 20CRv3. (**c**) Zoomed in panel on ERA20C. (**d**) Zoomed in panel on ERA5. (**e**) Zoomed in panel on NCEP1.

The above analysis establishes the usability of reanalysis data to assess changes in atmospheric water fluxes and temperature. It also highlights the different insight gained from $P - E$ and $P + E$. We will now unveil further details through a graphical framework that integrates precipitation, evaporation, their difference, and their sum^24^. By transforming the changes in the relationship of *P* and *E* to changes in $P - E$ and $P + E$, we can describe the water cycle dynamics in terms of atmospheric water storage and fluxes correspondingly. We apply this procedure to the four reanalyses to explore their representation of water cycle between two 30-year periods (1951–1980 and 1981–2010; Fig. 6). It is easy to pinpoint some distinguishable features for each data set. The 20CR v3 appears to have substantially higher atmospheric water flux estimates than any other reanalysis. However, if we decompose it in $P - E$ and $P + E$ terms, we can see that in the two periods examined, the difference between precipitation and evaporation increased (blue vector), implying atmospheric water loss (Fig. 6b). In ERA5, the exact opposite behavior emerges. The atmospheric water content has been increasing, but overall the average conditions suggest that the atmosphere has been getting drier since 1950 (Fig. 6d). The remaining two reanalyses manifest a stationary relationship in the water storage with no changes in the $P - E$ component (Fig. 6c,e). Surprisingly, the flux of atmospheric water is decreasing in NCEP1, suggesting a weakening of the water cycle (green arrow; Fig. 6c).

It is evident that no two reanalyses are alike when it comes to the exchange of water between the land-ocean continuum and the atmosphere at the global scale. In terms of magnitude, ERA5 reports changes in $P + E$ accelerating almost twice as fast as in the 20CR v3 and ERA-20C (41.5 mm/year versus 23.69 mm/year and 25.3 mm/year, respectively). The $P + E$ change in NCEP1 is similar to that observed in the 20CR v3 and ERA-20C. Although as already mentioned, in the opposite direction. Looking beyond 1950, in the reanalyses with longer records (20CR v3 and ERA-20C), we can see an agreement in the direction of change since 1921. Additionally, both reanalyses show a higher increase in $P + E$ between 1951–1980 and 1981–2010 than between 1921–1950 and 1951–1980. What is different, though, is the behavior of $P - E$, especially if analyzed over their 30-year average trajectory (Fig. 6b,c, light gray points). In ERA-20C, $P - E$ changes remain consistently stationary and very close to zero (0.15 mm/year), while in the 20CR v3 oscillates substantially following both increasing and decreasing patterns over the last 120 years. The trajectories of the other two reanalyses show behaviors somewhere in between, with more flexibility in $P - E$ compared to ERA-20C but not as much freedom as in the 20CR v3. Overall, the combination of $P - E$ and $P + E$ revealed a wealth of additional information about the reanalyses performance that is easily communicable and reproducible through the precipitation–evaporation space graphical framework, shaping the path for further investigations into the reasons behind these differences.

## Discussion

Due to the lack of robust observational-based data for crucial water cycle components such as evaporation, reanalyses data is still one of our best tools for researching changes in the global water cycle. The results fall within persistent criticism toward reanalyses (e.g., substantial variability^5^ and overestimations^7^) but advocate for the framework proposed to acquire new insight and improve climate reanalysis. We displayed how while $P - E$, a key diagnostic, is not directly observable at the global scale, $P + E$ is not held back by scale limitations and complements global water cycle research. Most significantly, including $P + E$ revealed additional information about the water cycle changes characteristics in four reanalyses. Information that could be implemented to address non-physical trends and inhomogeneities due to changes in the observing system (e.g.,^29^) and water budget non-closure (e.g.,^30,31^). The latter is an ongoing challenge in global water cycle research^32^, and non-closure is present in all reanalyses. Unexpectedly, although, we found a spurious long-term correlation between $P - E$ and temperature, suggesting such an artifact might be rooted in model processes and not only due to assimilation schemes. Along that line, we were surprised to find that the correlation between evaporation and temperature is smaller than that between precipitation and temperature in reanalyses, except for ERA5.

Needless to say, a persistent challenge is the unconstrained uncertainty in quantifying water cycle fluxes. Of particular relevance herein is that global $P - E$ is small, and its uncertainty might easily be much larger than its value. Thus, the signal-to-noise ratio of changes in $P - E$ versus the natural variability will be low and as a consequence the fluctuations in water cycle harder to detect. This limitation can be overcome when using $P + E$, which is less prone to the reanalyses uncertainties. These uncertainties could be encapsulated by their assimilation scheme, considering the assimilation scheme includes, among others: the forecast model, boundary conditions, observations, observation operators, and covariance models^33^. Put simply, differences in reanalysis assimilation schemes can significantly impact precipitation and evaporation inherent uncertainties. NCEP1 reanalysis uses a 3D-Var data assimilation system, which minimizes the difference between the model and observations by adjusting the atmospheric state variables^15^. On the other hand, ERA-20C and ERA5 use a 4D-Var data assimilation system, which adjusts the atmospheric state variables over a series of time steps to minimize the difference between the model and observations^13,14^. The 20CR v3 uses a hybrid 4D-Var/Ensemble Kalman Filter data assimilation system, which combines the strengths of both 3D-Var and 4D-Var to improve the accuracy of the precipitation and evaporation estimates^12^. Although it is not the scope of this study to address the underlying uncertainties or the effects of different assimilation schemes, looking into discrepancies among reanalyses estimates offers a handy demonstration of what can be learned by utilizing the precipitation–evaporation space to assess water cycle changes.

Whilst some features were common for all or most reanalyses, like changes in $P - E$ being much smaller than in $P + E$ and an increase in $P + E$ between the two most recent 30-year periods, we observed various individual distinctions. Out of the four reanalyses, ERA5 had the most comprehensive representation of precipitation and temperature variability compared to observational-based references, and was found to represent better the acceleration dynamics between 1951–1980 and 1981–2010. At the same time, ERA5 has the most pronounced changes for $P - E$, showcasing improvements in its terrestrial water storage computations^34^. However, ERA5 has the steepest acceleration of $P + E$ and is the only reanalysis above the $P = E$ isoline for the entirety of its record, which could be an artifact attributed to precipitation overestimations identified across different regions^35^. To the opposite end, NCEP1 shows a decline in atmospheric water fluxes over time with a slight decrease in atmospheric water storage. Forbye, the 30-year average trajectory exhibits an acute u-turn between the mid-1960s and the late 1970s. Around this trajectory inversion, the behavior is similar to ERA-20C with little to no variability along a $P - E$ isoline. A possible explanation for this abnormal behavior could be traced back to remote sensing data assimilation. Inconsistencies in its atmospheric data pre-1979 have previously been reported and associated with the lack of satellite observations before 1979, e.g., in the Southern Hemisphere^36^.

Using solely $P + E$ comes with its own limitations and could mask the true dynamics of global water cycle change. The reciprocal complementarity of $P + E$ and $P - E$ is better perceived on the long-record reanalyses. The overview clearly shows that the 20CR v3 portrays a warmer and wetter Earth relative to the rest of the reanalyses. This is consistent with a systematic bias in tropical precipitation^12^, and biases in the vertical structure of mass and circulation determined throughout the atmosphere^37^. Having said that, the magnitude of changes in $P + E$ are consistent with those of ERA-20C. The most recent increase is higher than the preceding ones and suggests that the global water cycle acceleration signal has further strengthened in the last three decades^38^. The above would suggest that changes in the global water cycle are similarly represented on both data sets. In sooth, $P - E$ changes in the 20CR v3 oscillate substantially following both increasing and decreasing patterns, whereas ERA-20C shows little to no variability (no spurious jumps or trends) and steadily moves along a $P - E$ isoline. Said stability lies around water cycle budget non-closure because evaporation is higher than precipitation despite known systematic precipitation overestimation^39^. Reportedly, there are only subtle differences in the data assimilated and the data assimilation schemes between these two reanalyses^13^, yet we can see contrasting behaviors exposed within the framework proposed herein.

Our findings, including the good agreement with the range of hydrological sensitivity, advocate for the definitions of $P + E$ to be physically sound. It is important, nonetheless, to note that such an agreement is not a two-way relationship. As seen in our examination, the fact that all the reanalyses have similar hydrological sensitivities does not necessarily mean that they express a similar rate of water cycle changes. Assuming so could be misleading, whereas we can get more insight and avoid these pitfalls by decomposing the change into $P - E$ and $P + E$ (i.e., into water storage and fluxes). It could be argued that introducing a new metric for acceleration into the current broad spectrum of metrics may lead to inconsistent hydroclimatology analysis terminology, such as that recently reported for wetter and drier^40^. Nevertheless, $P + E$ is not just an index because it is physically grounded and, as such, is better suited to describe climate models and reanalyses^41^.

Along the same line, it could be argued that assessing changes in precipitation or evaporation alone can directly indicate changes in the water cycle. It is easy to imagine that altering the state of one component in the water cycle would affect the others. However, the global water cycle is a complex phenomenon composed of several processes that we are yet to understand fully. Hence, changes in one component might not be instantly observed in the others. The compound behavior of precipitation and evaporation provides a more comprehensive picture of the water balance because it considers both the supply and demand of water or, within the precipitation–evaporation space, both atmospheric water storage ($P - E$) and water cycle intensity ($P + E$). As evinced by our results, precipitation increases are evident in all reanalyses. Regardless, until we inspect these reanalyses in the precipitation–evaporation space, we cannot observe that, in reality, no reanalysis is alike as they all describe different water cycle dynamics.

The above applications highlight the potential of $P + E$ to complement water cycle research at the global scale. The proposed framework could advance our understanding of water cycle changes and improve climate modeling. We have already revealed some discrepancies between the reanalysis data sets. Still to properly address them, the observational limitations at global scale, especially in evaporation, need to be overcome^42^. Additionally, it is intriguing to see how the total water transfer between the land-ocean continuum and atmosphere appears in Earth System Models and whether it can be also applied as a metric for the model performance. Future research into global spatial patterns of $P + E$ could also shed more light on how they relate to regional changes and hydroclimatic extremes such as droughts. To this extent, quantifying the surface-atmosphere water exchange in the form of $P + E$ can enhance our insight into past, present, and future hydroclimatic variability.

## Methods

### Data

We selected four reanalysis data products (Table 3). These are the Twentieth Century Reanalysis (20CR) v3^12^, European Centre for Medium-Range Weather Forecasts (ECMWF) Reanalyses ERA-20C^13^ and ERA5^14^, and the National Centers for Environmental Prediction & the National Center for Atmospheric Research NCEP/NCAR Reanalysis 1^15^. The 20CRv3 and the ERA-20C have two of the longest record among reanalyses, with 180 and 100 years, respectively. ERA5 and NCEP1 are two distinctive ongoing projects. ERA5 is a fifth-generation reanalysis (the most recent to date), and NCEP1 is a first-generation reanalysis. NCEP1 it is the longest-running reanalysis that uses rawindsonde data, but the model and data assimilation scheme are antiquated^7^. Notwithstanding, the Climate Prediction Center (CPC) Merged Analysis of Precipitation (CMAP) data set^43^, which is highly regarded as an observational-based reference^44^, blends NCEP1 to fill missing data.

Additionally, we used two observation-based products. For precipitation, the Global Precipitation Climatology Project (GPCP) v2.3^16^, which merges data from rain gauge stations, satellites, and sounding observations. For temperature, the HadCRUT5^17^ from the Met Office Hadley Centre and the Climatic Research Unit at the University of East Anglia, which blends data from meteorological stations, ships, and buoys. All of the above data sets are available for download on the dedicated websites of their providers. Through the pRecipe R package (https://cran.r-project.org/package=pRecipe), we computed the area-weighted average of gridded data and generated annual time series for total atmospheric water fluxes and global mean temperature.

**Table 3:** Data set overview.

Name	Source	Model resolution	Record length	Assimilation schemes	References
20CR v3	NOAA	T254 ($\approx 75$ km at the equator)	1836–2015	Ensemble Kalman Filter and 4-dimensional incremental analysis update (EnKF-4DIAU)	^12^
ERA-20C	ECMWF	T159 ($\approx 125$ km at the equator)	1900–2010	4-dimensional variational assimilation (4D-Var)	^13^
ERA5	ECMWF	T639 ($\approx 31$ km at the equator)	1950–now	4-dimensional variational assimilation (4D-Var)	^14^
NCEP1	NCEP NCAR	T62 ($\approx 210$ km at the equator)	1948–now	3-dimensional variational assimilation (3D-Var)	^15^

### Benchmarking reanalyses

We examined some commonly used statistical metrics to benchmark the reanalysis data products. Their aptness to capture the temporal variability of the water cycle was quantified via:

- The square of the Pearson correlation coefficient (R-squared or $R^{2}$)
  $$\begin{aligned} R^{2} = 1 - \frac{\sum _{i}^{n} \left( y_{i} - \hat{y}_{i}\right) ^{2}}{\sum _{i}^{n}\left( y_{i} - {\overline{y}}\right) ^{2}} \end{aligned}$$
  where *i* starts on the first year of the available record, *n* is the last year of the available record, $y_{i}$ is the observational estimate on year *i*, $\hat{y}_{i}$ is the reanalysis estimate on year *i*, and ${\overline{y}}$ is the mean observational estimate for the full available record.
- Root Mean Square Error (RMSE)
  $$\begin{aligned} \text {RMSE} = \sqrt{\frac{\sum _{i}^{n} \left( y_{i} - \hat{y}_{i}\right) ^{2}}{N}} \end{aligned}$$
  where *i* starts on the first year of the available record, *n* is the last year of the available record, $y_{i}$ is the observational estimate on year *i*, $\hat{y}_{i}$ is the reanalysis estimate on year *i*, and *N* is the total number of years in the full available record.

Only precipitation and temperature records were evaluated because there is no robust observation-based evaporation data set. Note that precipitation and temperature were compared using two different reference periods because GPCP v2.3 record starts in 1979, and HadCRU5T5 provides only temperature anomalies using the 1961–1990 average as a reference. Thus, we could not homogenize the reference period for both variables and selected 1981–2010 for precipitation and 1961–1990 for temperature. Subsequently, we inspected global water budget closure via the 30-year moving average of $P - E$.

### Thermodynamics of atmospheric fluxes

For superimposing the temperature to the precipitation plus evaporation time series, without incurring in any kind of data tampering, we simply rescaled temperature to precipitation plus evaporation in the same way one would rescale degrees Fahrenheit to degrees Celsius. I.e.:

$$\begin{aligned} y'_{i} = \left( \left( T_{i} - min(T)\right) *\frac{max(P + E) - min(P + E)}{max(T) - min(T)}\right) + min(P + E) \end{aligned}$$

where $y'_{i}$ denotes the value used to plot Temperature in the same scale of precipitation plus evaporation for any given year, $T_{i}$ is the temperature reanalysis estimate on year *i*, *min*(*T*) is the minimum temperature reanalysis estimate in the full available record, $max(P + E)$ is the maximum precipitation plus evaporation reanalysis estimate in the full available record, $min(P + E)$ is the minimum reanalysis estimate in the full available record, and *max*(*T*) is the maximum temperature reanalysis estimate in the full available record.

As thermodynamics dictates, we expect a linear relationship between atmospheric water fluxes and temperature. This correspondence was quantified via the square of the Pearson correlation coefficient (R-squared or $R^{2}$)

$$\begin{aligned} R^{2} = 1 - \frac{\sum _{i = 1}^{n} \left( y_{i} - f(T_{i})\right) ^{2}}{\sum _{i = 1}^{n}\left( y_{i} - {\overline{y}}\right) ^{2}} \end{aligned}$$

where *n* is the total number of years in the full available record, $y_{i}$ is the i-th reanalysis estimate for atmospheric water flux, $f(T_{i})$ is the i-th predicted estimate by temperature, and ${\overline{y}}$ is the mean reanalysis estimate for the full available record. The same metrics were computed again for the annual differences of each time series (i.e., $\delta (y_{i}) = y_{i} - y_{i - 1}$). To this extent, we can characterize the long-term and the year-to-year association between atmospheric water fluxes and temperature. While the correlation coefficient describes the presence or absence of a linear relationship, it does not quantify the rate of change of one variable relative to the other. Henceforth, we used linear regression to estimate the corresponding slopes and describe the rate of change at which atmospheric water fluxes respond to changes in temperature. To compare the slopes between data sets on a one-to-one basis, we estimated atmospheric water fluxes and temperature in terms of global anomalies with respect to the 1981–2010 period.

$$\begin{aligned} \text {slope} = \frac{n\left( \sum _{i = 1}^{n}T_{i}y_{i}\right) - \left( \sum _{i = 1}^{n}T_{i}\right) \left( \sum _{i = 1}^{n}y_{i}\right) }{n\left( \sum _{i = 1}^{n}T_{i}^{2}\right) - \left( \sum _{i = 1}^{n}T_{i}\right) ^{2}} \end{aligned}$$

where *n* is the total number of years in the full available record, $y_{i}$ is the i-th reanalysis estimate for atmospheric water flux anomaly, and $T_{i}$ is the i-th reanalysis estimate for temperature anomaly. Lastly, we relied on the Residual Standard Error (RSE) to assess the goodness-of-fit of the slopes, i.e., how well these slopes represent the linear relationship between our variables.

$$\begin{aligned} \text {RSE} = \sqrt{\frac{\sum _{i = 1}^{n} \left( y_{i} - f(T_{i})\right) ^{2}}{n - 1}} \end{aligned}$$

where *n* is the total number of years in the full available record, $y_{i}$ is the i-th reanalysis estimate for atmospheric water flux anomaly, and $f(T_{i})$ is the i-th predicted estimate by temperature anomaly.

## Data Availability

The precipitation, evaporation, and temperature global reanalysis data described in Table 3 were downloaded from the KNMI Climate Explorer at https://climexp.knmi.nl. The annual time series were generated via area weighted averaging using the *pRecipe* R package, which is preserved at https://cran.r-project.org/package=pRecipe.

## Code availability

The data generated herein and the R scripts for the figures presented in the study are publicly available at https://github.com/MiRoVaGo/P_plus_E, and it is preserved at https://doi.org/10.5281/zenodo.7299251.
